# Old Cures for Hydrophobia

**Published:** 1889-12-28

**Authors:** 


### Old Cures for Hydrophobia.

"M. D." writes: "I have copied the enclosed from bits of old newspapers, which I cut off some twenty-five or thirty years ago. Please do with them what you think best":

Dr. Buisson, says the *Salut Public*, of Lyons, claims to have discovered a remedy for this terrible disease, and to have applied it with complete success in many cases. In attending a female patient in the last stage of canine rabies, the doctor imprudently wiped his hands with a handkerchief impregnated with her saliva. There happened to be a slight abrasion of the index finger of the left hand, and confident in his own curative system, the doctor merely washed the part with water. However, he was fully aware of the imprudence he had committed, and gives the following account of the matter afterwards: "Believing that the malady would not declare itself until the fortieth day, and having numerous patients to visit, I put off from day to day the application of my remedy—that is to say, vapour baths. The ninth day, being in my cabinet, I felt all at once a pain in the throat, and a still greater one in the eyes. My body seemed so light that I felt as if I could jump to a prodigious height, or that if I threw myself out of a window I could sustain myself in the air. My hair was so sensitive that I appeared able to count each separately without looking at it. Saliva kept continually forming in the mouth. Any movement of air inflicted great pain on me, and I was obliged to avoid the sight of brilliant objects. I had a continual desire to run and bite, not human beings, but animals, and all that was near me. I drank with difficulty, and I remarked that the sight of water distressed me more than the pain in the throat. I believe that by shutting the eyes anyone suffering under hydrophobia can always drink. The fits came on every five minutes, and I then felt the pain start from the index finger and run up the nerves to the shoulder. In this state, thinking that my course was preservative and not curative, I took a vapour bath, not with the intention of cure, but of suffocating myself. When the bath was at a heat of 52 C. (93 3-5 F.) all the symptoms disappeared as if by magic, and since then I have never felt anything more of them. I have attended more than eighty persons bitten by mad animals, and I have not lost a single case." When a person has been bitten by a mad dog he must for seven successive days take a vapour bath *a la Russe*, as it is called, of 57deg. to 63deg. This is the preventive remedy. When the disease is declared it only requires one vapour bath, rapidly increased to 36 C., then slowly to 63. The patient must strongly confine himself to his chamber until the cure is complete. Dr. Buisson mentions several other curious facts. An American had been bitten by a rattlesnake about eight leagues away from home. Wishing to die in the bosom of his family, he ran the greater part of the way home, and, going to bed, perspired profusely, and the wound healed as any simple cut. The bite of the tarantula is cured by the exercise of dancing, the free perspiration dissipating the virus. If a young child be vaccinated, and then be made to take a vapour bath, the vaccine does not take.—*Galignani.*

